# Cell Cycle Heterogeneity Can Generate Robust Cell Type Proportioning

**DOI:** 10.1016/j.devcel.2018.09.023

**Published:** 2018-11-19

**Authors:** Nicole Gruenheit, Katie Parkinson, Christopher A. Brimson, Satoshi Kuwana, Edward J. Johnson, Koki Nagayama, Jack Llewellyn, William M. Salvidge, Balint Stewart, Thomas Keller, Wouter van Zon, Simon L. Cotter, Christopher R.L. Thompson

**Affiliations:** 1 Centre for Life’s Origins and Evolution, Department of Genetics, Evolution and Environment, University College London, Darwin Building, Gower Street, London WC1E 6BT, UK; 2 Division of Developmental Biology and Medicine, Faculty of Biology, Medicine and Health, The University of Manchester, Michael Smith Building, Oxford Road, Manchester M13 9PT, UK; 3 School of Mathematics, Faculty of Science and Engineering, The University of Manchester, Alan Turing Building, Manchester M13 9PL, UK

**Keywords:** heterogeneity, cell cycle, single-cell sequencing, cell fate, lineage priming, *Dictyostelium*, oscillator

## Abstract

Cell-cell heterogeneity can facilitate lineage choice during embryonic development because it primes cells to respond to differentiation cues. However, remarkably little is known about the origin of heterogeneity or whether intrinsic and extrinsic variation can be controlled to generate reproducible cell type proportioning seen *in vivo*. Here, we use experimentation and modeling in *D. discoideum* to demonstrate that population-level cell cycle heterogeneity can be optimized to generate robust cell fate proportioning. First, cell cycle position is quantitatively linked to responsiveness to differentiation-inducing signals. Second, intrinsic variation in cell cycle length ensures cells are randomly distributed throughout the cell cycle at the onset of multicellular development. Finally, extrinsic perturbation of optimal cell cycle heterogeneity is buffered by compensatory changes in global signal responsiveness. These studies thus illustrate key regulatory principles underlying cell-cell heterogeneity optimization and the generation of robust and reproducible fate choice in development.

## Highlights

• *Dictyostelium* cells break symmetry in a stochastic salt and pepper fashion
• Cell cycle position affects responsiveness to differentiation inducing signals
• Cell cycle length variation ensures cells are distributed in different cycle phases
• Perturbation of cell cycle dynamics is buffered by changes in signal responsiveness

## Graphical Abstract

**Figure:**
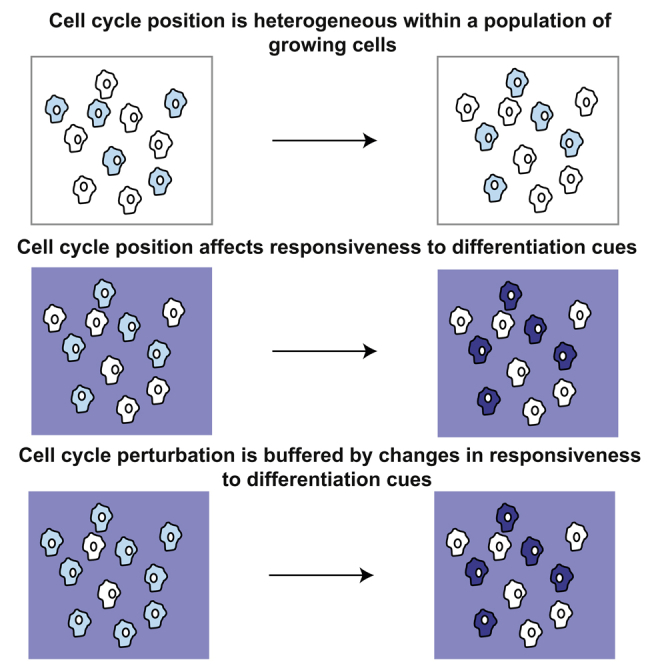

## Teaser

Gruenheit *et al.* find cell cycle position heterogeneity promotes symmetry breaking during development as it helps cells respond to different differentiation cues. Buffering by changes in signal responses ensures this system is robust to perturbation of cell cycle dynamics. This illustrates how heterogeneity can be optimized to facilitate development.

## Introduction

Tissue patterning and cell type proportioning are robust and reproducible. However, cell-cell heterogeneity can prevent cell populations from behaving in a coordinated fashion. Much research has focused on how this variation is tolerated. For example, many developmental systems are regulative and can correct errors (Lawrence and Levine, 2006). Gene networks underlying cell fate choice can also buffer fluctuations (Averbukh et al., 2017, Levy and Siegal, 2008). Recent studies, however, suggest that heterogeneity can increase the spectrum of differentiation capabilities of cells in a uniform environment (Altschuler and Wu, 2010, Balázsi et al., 2011). Most notably, this leads to random “salt-and-pepper” differentiation where reproducible proportions of different cell types still arise. Examples range from competence in *B. subtilis* (Maamar et al., 2007) to lineage specification in the mouse blastocyst (Dietrich and Hiiragi, 2007).

Although the molecular mechanisms underlying salt-and-pepper differentiation are poorly understood, general principles are emerging. First, heterogeneity is thought to prime some cells to adopt a particular lineage (Canham et al., 2010, Chang et al., 2008). For example, priming could affect the likelihood that a cell will respond to signals that trigger differentiation, even if all cells receive the signals (i.e., it affects the threshold of responsiveness) (Canham et al., 2010, Chang et al., 2008). Alternatively, in cases where differentiation is cell-autonomous and achieved in the absence of an external cue, primed cells may simply express different amounts of key regulators of the differentiation program (Maamar et al., 2007). Second, the primed state is thought to be unstable and transient (Canham et al., 2010, Filipczyk et al., 2015, Süel et al., 2006). For example, when primed cells are isolated and regrown, the heterogeneous population is rapidly reconstituted (Canham et al., 2010, Chang et al., 2008). Despite this emerging framework, it is unclear how the expression of lineage priming genes affects the threshold of responsiveness or cell fate choice at the molecular level. Furthermore, because few lineage priming genes have been identified, it is unknown how lineage priming dynamics or the number of lineage-primed cells is controlled. Addressing these questions will be crucial to understanding how this mechanism can achieve robust cell type proportioning.

Stochastic lineage priming dynamics provide one method of achieving robust developmental outcomes (Schultz et al., 2007). This is because even though the behavior of one cell may be unpredictable, the probability of a proportion of cells within a population being in a primed state can be fixed. Alternatively, there is evidence that lineage priming dynamics can be governed by an underlying oscillatory mechanism that reproducibly drives cells in and out of a primed state (Soufi and Dalton, 2016). For example, studies of human embryonic stem cell (hESC) differentiation have revealed a relationship between the cell cycle and lineage potential (Li and Kirschner, 2014, Pauklin and Vallier, 2013, Roccio et al., 2013). Differentiation of hESCs is favored in the G1 phase of the cell cycle, with endoderm fate favored in early G1 and neuroectoderm fate in late G1. However, induction of neuroectoderm and endoderm occurs in response to positional signals during gastrulation, rather than in a salt-and-pepper distribution (Rossant and Tam, 2009). Consequently, how these cell culture observations relate to differentiation *in vivo* is unknown. Despite these limitations, these studies demonstrate that cell cycle position allows an asynchronous population of cells to exhibit differential responses to a uniform stimulus and thus provides an attractive candidate mechanism for robust salt-and-pepper differentiation *in vivo*.

To address this possibility, we have utilized the social amoeba *Dictyostelium discoideum*. Individual *Dictyostelium* amoebae grow and divide when nutrients are present. However, when nutrient supplies are exhausted, approximately 10^5^ cells aggregate to form a multicellular mound, which undergoes a program of cell type differentiation and morphogenesis. It is thought that although growing cells are undifferentiated, they are dynamically and unstably primed toward future developmental fates (Chattwood et al., 2013). Differences in cell cycle position (Araki et al., 1994, Gomer and Firtel, 1987, Thompson and Kay, 2000a, Weijer et al., 1984), intracellular calcium concentration (Kubohara et al., 2007, Schaap et al., 1996), intracellular pH (Gross et al., 1983, Kubohara et al., 2007), and nutritional history (Leach et al., 1973, Thompson and Kay, 2000a) of growing cells have all been shown to bias cell fate choice and vary dynamically within populations of cells. Furthermore, it has been shown that these biases modulate the threshold of responsiveness to diffusible developmental signals, such as cAMP and DIF-1 (Chattwood et al., 2013, Thompson and Kay, 2000a), which only accumulate to high levels following starvation and aggregation (Kay and Thompson, 2001). Cell type differentiation thus takes place at the mound stage. DIF-1 promotes the differentiation of prestalk populations including ecmA-expressing pstAO cells and ecmB-expressing pstB cells (Keller and Thompson, 2008, Thompson and Kay, 2000b) (Figure S1). cAMP promotes the differentiation of prespore cells, which express the pspA gene. As all cells within the mound are uniformly exposed to these signals, it has been proposed that intrinsic differences in thresholds of responsiveness result in salt-and-pepper differentiation (Chattwood et al., 2013, Thompson et al., 2004b). Crucially, multicellular development in *Dictyostelium* is extremely robust, with cell type proportioning reproducible between multicellular aggregates and over a wide range of total cell numbers (Bonner, 1957, Ràfols et al., 2001). One possibility is that initial symmetry breaking is noisy but stabilized by feedback loops in which DIF-1 is synthesized by prespore cells and degraded by prestalk cells to ensure DIF-1 levels reach an equilibrium and maintain prestalk-prespore proportioning (Kay and Thompson, 2001). Alternatively, initial symmetry could be precise and result in near correct proportioning that only requires correction if the system is severely perturbed. We, therefore, reasoned that *Dictyostelium* was an ideal system to address whether variation in cell cycle position can provide sufficiently robust population-level information to generate reproducible cell type proportioning *in vivo*.

## Results

### Single-Cell RNA-Seq Reveals Cell Cycle Position as the Major Driver of Cell-Cell Gene Expression Variation

**Figure 1:**
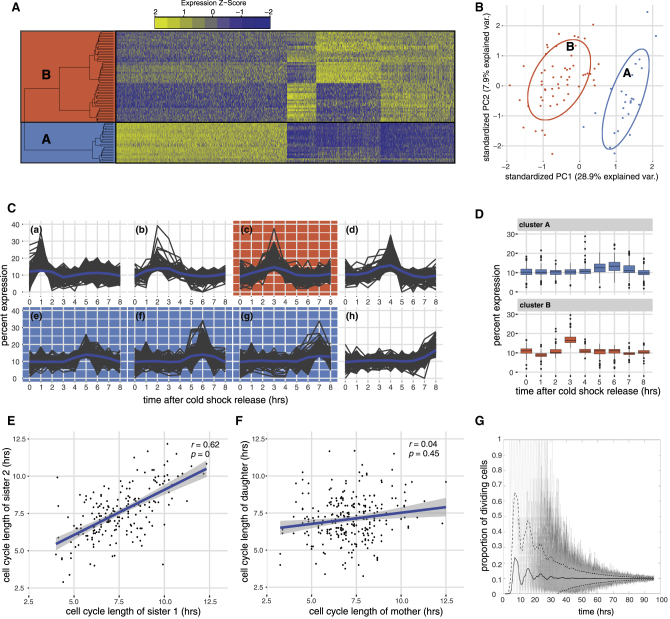
*Dictyostelium* Cells Exhibit Cell Cycle Heterogeneity (A) Gene expression heatmap for 1,602 marker genes reveals two clusters of vegetative cells. DEG analysis split the 81 vegetative cells into two groups. Expression values for each gene were mean-centered and scaled. (B) Principal-component analysis of 81 vegetative cells. The 500 most variable genes were used for PCA. Colors correspond to highlighted cells in (A). Normal data ellipse for each cluster is shown. (C) Cell cycle profiles of 6,228 genes. Cell cycle profiles from cold shock synchronized cells for 6,228 genes were extracted and normalized to percent expression (see STAR Methods). Genes that deviate more than 1.5 median absolute deviations (MADs) were binned according to the time point of their highest expression. Profiles for each bin were separated (a)–(h). The blue line denotes the mean. (D) Cell cycle profile of marker genes for cluster A and cluster B. A hypergeometric test was used to identify genes from cluster A (901 genes) and cluster B (701 genes) that were over-represented in the hourly cell cycle bins described in Figure 1C. Cluster A and cluster B genes show higher expression 5–7 and 3 hr after cold shock release, respectively. (E and F) Cell cycle length correlations. Cell cycle lengths of sister (E) or mother and daughter cells (F) were plotted. Blue line depicts the linear regression line (gray shaded area: 95% CI). (G) Cell divisions become rapidly de-synchronized in cell populations. Starting with one cell, 1,000 population simulations were run for 96 hr, and the number of cells dividing in each hour plotted (solid line: mean, dashed lines: 95% CI). The mean converges against a value of 0.11, which is indicative of de-synchronous cell cycle lengths.

In *Dictyostelium*, a clonal cell population can break symmetry and undergo robust cell fate choice and proportioning. We, therefore, reasoned that the major drivers of lineage priming should be detectable as variation in gene expression between growing cells. Moreover, we would expect the proportion of cells exhibiting distinct gene expression states to reflect the cell type proportions seen in development (the approximate ratio of prespore to prestalk cells is 70:30). To investigate this, we took an unbiased approach in which single-cell RNA sequencing (RNA-seq) was carried out on 81 log-phase vegetative cells to identify genes with highly variable expression patterns. Out of 11,319 expressed genes, we identified 1,619 significantly variable genes (p value = 0.01) by fitting a Michaelis-Menten model (K = 5.94). These differentially expressed genes (DEGs) revealed the presence of two large groups of cells, cluster A (25 cells) and cluster B (56 cells). For each gene, we next determined whether it could be used as a marker gene for cluster A or cluster B (or a subgroup thereof). Of the 1,619 DEGs, 1,602 genes had an AUC higher than 0.8, indicating that they are specific for either cluster A (901 genes; Figure 1A), or cluster B (701 genes; Figure 1A). 493 of these genes also show variation within cluster B, but expression within that cluster is still higher than the expression in cluster A. Because almost all significantly variable genes are also marker genes for these two clusters, this shows these genes are the main difference between the two groups. Finally, a principal-component analysis (PCA) using the 500 most variable genes also divided the cells into the same two groups (Figure 1B), and the percentage of cells in each group (31% and 69%) roughly corresponded to the ratio of prestalk and prespore cells seen during development.

To understand the molecular basis of this gene expression variation, we first performed Gene Ontology (GO) analyses. Cluster A genes showed enrichment for GO terms including “myosin II filament organization,” “mitotic cytokinesis,” and “cytokinesis” (Data S1). Marker genes for cluster B were enriched for GO terms including “oxidative phosphorylation,” “translation,” and various biosynthetic processes (Data S1). This raised the possibility that differences in cell-cycle-dependent gene expression could underlie the clusters, with cells in cluster A preparing to divide, undergoing, or having just undergone mitosis (M/S phase) and cells in cluster B actively growing (G2 phase). To test this, we used cells synchronized by cold shock to determine the *Dictyostelium* cell-cycle-dependent transcriptome. Cold shock likely arrests cells in late G2, because most cells undergo mitosis within 2–3 hr after cold shock release (Maeda, 1986, Strasser et al., 2012). BrdU incorporation reveals these cells then directly enter S phase (there is little or no G1 in *Dictyostelium*). Indeed, genes associated with M/S phase are up-regulated shortly after cold shock release (Strasser et al., 2012). Cells then undergo G2, with the second round of less synchronous mitosis typically observed around 8 hr after cold shock release. We used gene expression data from cells collected at hourly time points after cold shock release to capture one complete cell cycle. After filtering to remove poorly expressed genes, the remaining 6,121 genes were screened for significant expression peaks at different time points (see STAR Methods) and binned accordingly (Figure 1C and Data S2). GO analyses revealed strong cell cycle signatures, e.g., a significant over-representation of the terms “DNA replication,” “chromosome segregation,” and “nuclear division” 1 hr and 5–7 hr after cold shock release, which supports the idea that the majority of these cells are indeed in M/S phase or preparing to undergo mitosis (Data S2). Most importantly, using a hypergeometric test to identify over-representation, we found that cluster A genes were significantly enriched for genes with peaks in late G2 (p < 0.05; 5–7 hr after cold shock release) and then fall sharply after S phase (8 hr) (Figure 1D). It is also interesting to note that only a small number of cluster A genes have increased transcription 1 hr after cold shock release, which likely reflects the fact that cold shock specific perturbations affect the first M/S phase these cells undergo. The second M/S phase after cold shock therefore likely better reflects a normal cell cycle. Markers of cluster B, on the other hand, were significantly enriched for genes that peak 3 hr after cold shock release (p = 3.24 × 10^−31^; Figure 1D). For example, most ribosomal proteins exhibited this transcription pattern, which is indicative of cell growth. Consequently, our single cell analyses reveal that gene expression variation between individual *Dictyostelium* cells is likely a consequence of a strong cell cycle signature.

### Stochastic Cell Cycle Variation Results in Robust and Rapid De-synchronization of *Dictyostelium* Cell Populations

If cell cycle position underlies cell fate decisions in *Dictyostelium*, for proportioning to be reproducible, cells must be predictably distributed (on a population scale) throughout the cell cycle when differentiation is induced. Consequently, the *Dictyostelium* cell cycle must exhibit sufficient stochastic cell-cell variation to drive rapid population asynchrony and robust convergence to a steady-state distribution of cells in different positions in the cell cycle. To test this, we used time-lapse microscopy to measure cell cycle variation. This revealed sister cell divisions are correlated yet still somewhat variable (Figure 1E). However, no correlation was observed between mother and daughter cell cycle length (Figure 1F). Using these data, we next built a mathematical simulation to determine the number of cell divisions that would be required to completely de-synchronize a population of *Dictyostelium* cells that arises from a single cell (see STAR Methods). This revealed de-synchronization would occur within 40 hr, or five cell divisions (i.e., 32 cells) (Figure 1G). A single fruiting body is typically composed of more than 10,000 cells, and fruiting bodies of less than 1,000 cells can only be generated under very specific laboratory conditions (Konijn and Raper, 1961). This suggests synchrony would be lost long before cell differentiation. Therefore, if cell cycle position determined cell fate, the decision made by each cell would be unpredictable because it is based on a stochastic process. However, the average behavior of the population, due to the fast convergence to a steady-state cell cycle position distribution, would result in precise cell fate proportions.

### Cell Cycle Position Results in Oscillatory Cell Fate Choice Probabilities

**Figure 2:**
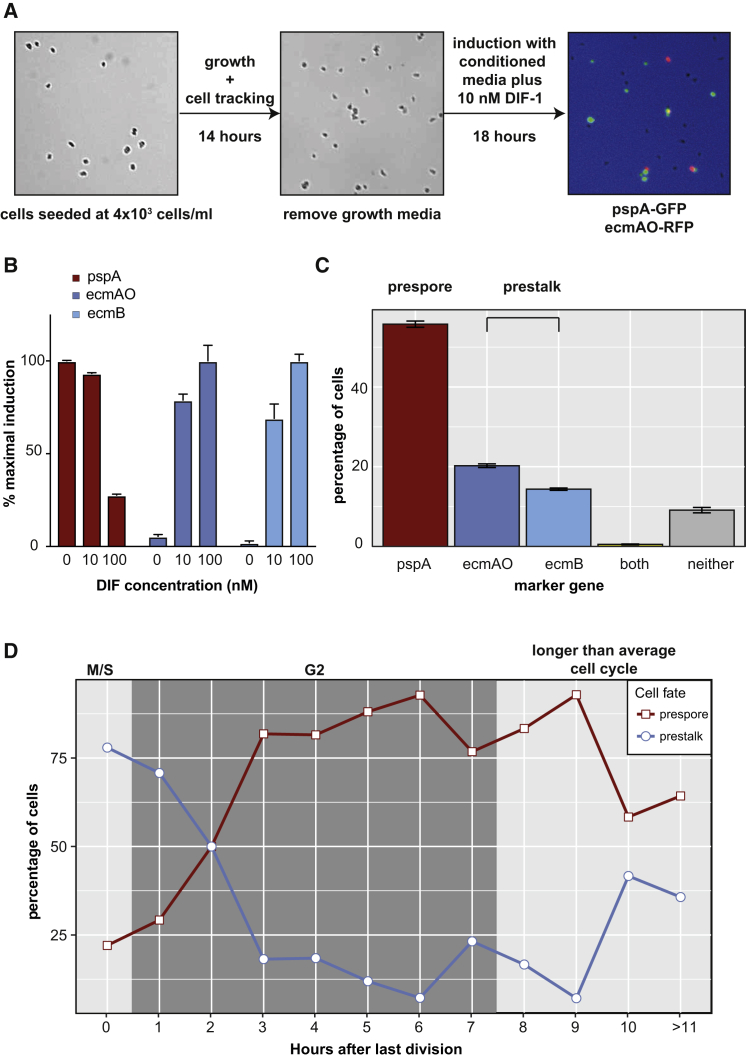
A Probabilistic Relationship between Cell Fate and Cell Cycle Position (A) Schematic of high-throughput, low-density growth and differentiation assay. (B) Dose-responsive induction and repression of prestalk and prespore marker gene expression. AX3 cells transformed with *pspA-RFP*, *ecmAO-RFP*, *or ecmB-RFP* cell type-specific reporters were incubated with indicated DIF-1 concentrations, and number of expressing cells determined by FACS. (C) Cell type differentiation at 10-nM DIF-1. AX3 cells co-transformed with *pspA*, *ecmAO*, or *ecmB* cell type specific reporters were incubated with 10-nM DIF-1 and the number of expressing cells determined by FACS (see STAR Methods). (D) Cell type differentiation is cell-cycle-dependent. 448 cells were tracked during growth and differentiation. Percentages of cells that differentiated as prespore or prestalk cells (ecmAO or ecmB expressing) were calculated for cells dividing within each hourly time interval before induction. The likely cell cycle position is highlighted.

To address whether cell cycle position is quantitatively linked to cell fate, we developed a high-throughput live imaging method to track cells during vegetative growth and differentiation (Figure 2A). Differentiation was induced by starvation for 18 hr in conditioned medium containing a cocktail of differentiation-inducing signals. To validate this method, fluorescence-activated cell sorting (FACS) quantification was used to determine the number of cells expressing markers of the major prespore (pspA-GFP) and prestalk (ecmAO-RFP or ecmB-RFP) cell types. As expected, the markers showed a dose-dependent response to the prestalk inducer DIF-1 (Figure 2B). Furthermore, at a dose of 10 nM DIF-1, which is thought to be close to physiological levels, the number of prestalk and prespore cells was similar to those seen *in vivo* (16% ecmB, 22% ecmAO, and 62% pspA expressing) (Figure 2C). For most cells, it was possible to unambiguously assign cell fate because less than 1% of the cells co-expressed two markers (Figure 2C). Finally, most cells differentiated, and less than 10% of the cells expressed no marker (Figure 2C).

To determine whether there is a relationship between cell cycle stage and prestalk or prespore fate choice, growing cells were filmed at low density for 12–14 hr. Under these conditions, cells have an average cell cycle length of 8.3 hr, and all cells can be tracked through at least one cell division (Video S1). Furthermore, analysis of PCNA-RFP expressing cells allowed us to confirm that the timing of the last cell division can be used to predict cell cycle position accurately. Almost all cells that underwent mitosis accumulated a PCNA-RFP dot in the nucleus within 20 min (76% within 20 min, 98% within 30 min; n = 93 cells) (Video S2). BrdU incorporation has previously revealed this coincides with late S phase (Muramoto and Chubb, 2008). This confirms previous observations that *Dictyostelium* cells have little or no G1 and that cells immediately enter S phase after mitosis, before cells enter G2. Once the growth medium was removed, and cell type differentiation induced, little further cell division was observed (6% of cells). Consequently, the timing of the last division relative to the addition of signals was used to define the cell cycle position of each cell when induced to differentiate. Finally, after 18 hr, a fluorescence image was taken to determine whether each cell had adopted a prespore (pspA-GFP) or prestalk (no fluorescence) fate (Video S1).

Video S1. Example Movie of Low-Density Growth and Differentiation Assay, Related to Figure 2

Video S2. Example Movie of PCNA Subcellular Localization during Low-Density Growth, Related to Figure 2

**Figure 3:**
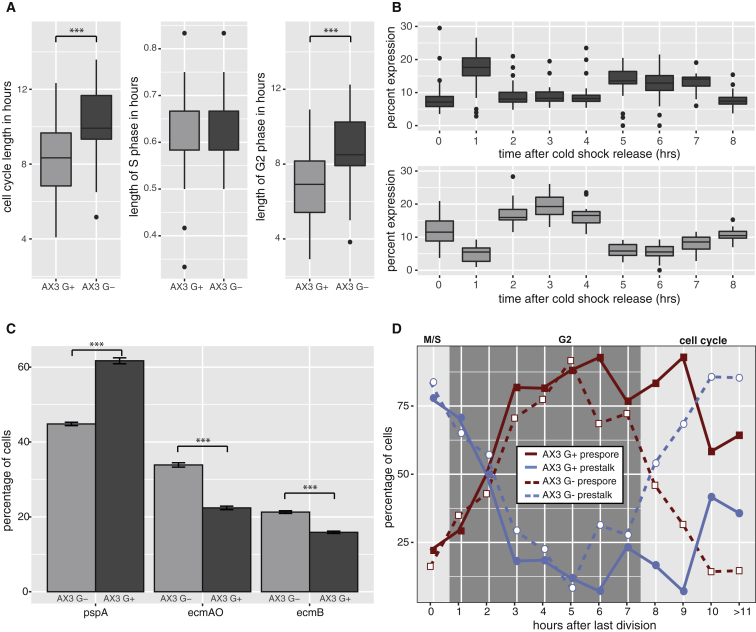
G− Growth Affects Cell Fate Choice due to Changes in Cell Cycle Dynamics (A) G− cells have a longer G2 phase. S and G2 phase lengths were determined from PCNA localization (Muramoto and Chubb, 2008). Only total cell cycle length and G2 phase length of G−-grown cells are significantly longer. (B) Differentially expressed genes between G+ and G− cells exhibit strong cell cycle profiles. A hypergeometric test was used to identify over-representation of DEGs between AX3 G+ and AX3 G− in the hourly cell cycle profiles (Figure 1C). (C) G− cells are biased toward prestalk cell fate. The percentage of prestalk and prespore cells was significantly different in G+ and G− cells in the low-density differentiation assay (t test; p = 5.5 × 10^−8^; p = 6 × 10^−7^; p = 3 × 10^−7^). (D) Stalk cell propensity increases in G− cells with a longer than average cell cycle length. Percentages of prespore and prestalk cells were plotted for G+ cells (solid line; see Figure 2D) and G− cells (dashed line) at different cell cycle positions. G− cells with a longer than average cell cycle (>8 hr) showed a much higher propensity to differentiate into prestalk cells.

Cell type differentiation was found to follow an oscillatory pattern. Prespore cell differentiation peaked in cells that underwent mitosis 6–7 hr before removal of growth medium (i.e., induced to differentiate in mid G2) (Figure 2D). The probability of prestalk cell differentiation was highest when cells had just undergone mitosis (i.e., in S phase). Prestalk or prespore cell differentiation was not restricted to specific cell cycle phases. For example, significant numbers of prestalk cells differentiated when cells divided 2–3 hr before the addition of differentiation signals. These cells cannot be in S phase because PCNA-RFP localization measurements reveal S phase length to be 37 min on average (μ = 37.4 min, σ = 5.1 min) and a maximum of 50 min (Figure 3A). Finally, cells at the end of G2 were also slightly stalkier than cells in the rest of G2 (Figure 2D). This suggested that the probability of prestalk cell differentiation increases in G2 but only just before cells enter mitosis. Indeed, cells with a significantly longer than average cell cycle length, which are more likely to divide within the next hour, are relatively stalky. Together, these findings suggest that the cell cycle acts as a probabilistic cell fate oscillator that regulates the threshold of responsiveness to differentiation signals.

### Nutritional History Affects Cell Cycle Length and Cell Fate Choice

**Figure 4:**
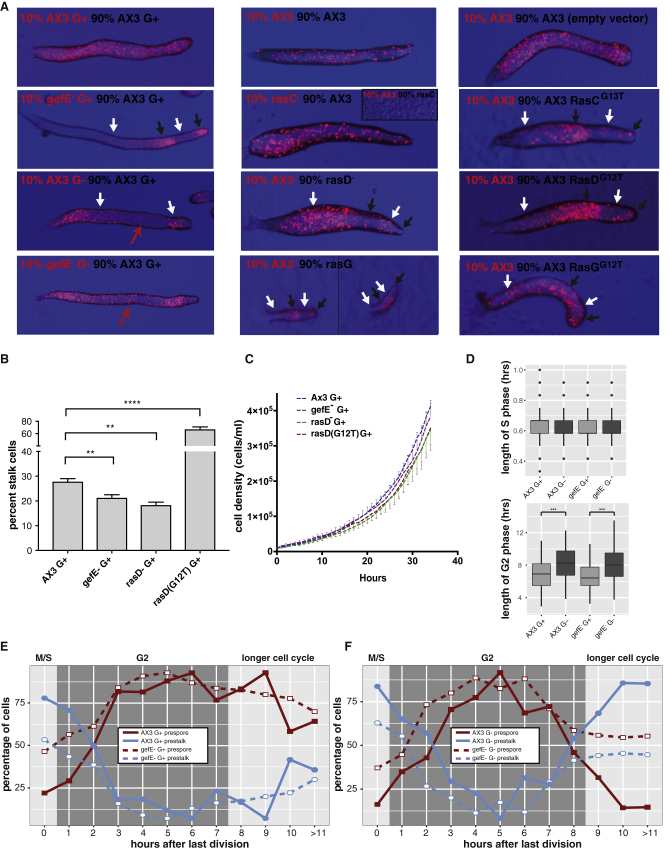
RasD Activity Affects Cell Fate Choice but Not Cell Cycle Dynamics (A) Ras activity and nutrition affect cell fate choice in chimeric development. Left panel: *gefE*^−^ cells exhibit a prespore and tip bias (black arrows) in chimera with wild-type cells. G− cells predominantly sort to the collar and back in chimera with G+ cells. *gefE*^−^ G− cells are also found in the spore region (red arrows), which results in partial rescue of the G− effect (Chattwood et al., 2013). Middle panel: labeled wild-type cells were mixed with unlabeled *rasC*^−^, *rasD*^−^, or *rasG*^−^ cells. *rasD*^−^ cells exhibit a sporey bias (black arrows), forcing wild-type cells to adopt prestalk fates (white arrows). *rasG*^−^ cells exhibit a stalky bias. *rasC*^−^ cells do not develop when in the majority. They co-aggregate when wild-type cells are in the majority but do not show a cell fate preference. Right panel: labeled wild-type cells were mixed with unlabeled cells induced to express constitutively active rasC^(G12T)^, rasD^(G12T)^, or rasG^(G12T)^. rasD^(G12T)^ expression resulted in the strongest effect, forcing cells toward the collar and back fate (i.e., opposite to the *rasD*^−^ or *gefE*^−^ mutant phenotype). (B) RasD activity and nutrition affect stalk cell differentiation in monolayer cultures. Decreased Ras activity (*rasD*^−^ or *gefE*^−^) significantly reduced stalk cell differentiation at 10-nM DIF-1 (3 replicates). In contrast, increased RasD activity (AX3^rasD^[G12T]) significantly increased the percentage of prestalk cells (t test: p = 0.0088; p = 0.0012; p = 0.0001). (C) Cell cycle length is not affected by RasD activity. The growth rate of wild-type AX3, *gefE*^−^, *rasD*^−^ mutant, and RasD^G12T^ cells was measured in culture. Error bars are standard error of mean of 3 replicates. (D) Cell cycle phase lengths are unaffected by RasD activity. PCNA localization was used to determine S and G2 length. There is no significant difference in S phase lengths, whereas total cell cycle and G2 phase lengths of G−-grown cells in wild-type and *gefE*^−^ are significantly longer. (E and F) Stalk cell propensity decreases in *gefE*^−^ G+ and *gefE*^−^ G− cells. *gefE*^−^ G+ cells (E) that divided up to 2 hr before induction have a higher propensity to become prespore cells. *gefE*^−^ G− cell (F) propensity to become prespore cells is higher than AX3 G− cells at almost all time points or cell cycle phases.

Our findings suggest that the *Dictyostelium* cell cycle is an example of a dynamic system that relies on stochasticity (in cell cycle length) to ensure robust cell type proportioning. One problem, however, is that extrinsic disruption of population heterogeneity, oscillator period, or amplitude would have a deleterious effect on development. Indeed, extrinsic environmental perturbations, such as pH, temperature, or metabolic changes, affect cell fate choice. However, it is unknown whether these effects are due to changes in cell cycle dynamics or other changes in cell physiology. To test this, we further studied the behavior of cells grown in media containing low glucose (G−), which are biased toward the prestalk cell fate (Leach et al., 1973, Thompson and Kay, 2000a; Figure 4A). We found G− cells have a significantly longer cell cycle than G+ cells (t test; p = 6.18 × 10^−6^; Figure 3A). However, not all cell cycle phases increase in length. PCNA-RFP localization revealed no difference in M/S phase length (t test; p = 0.56; Figure 4A), whereas G2 was significantly lengthened (t test; p = 6.25 × 10^−6^; Figure 3A). This finding initially appeared counterintuitive because mid-G2 cells tend to differentiate as prespore cells. However, our single cell analyses did reveal that the small number of G+ cells with a significantly longer than average cell cycle are biased toward the prestalk cell fate (Figure 2D). This raised the possibility that G− grown cells (and G+ cells with a longer than average cell cycle length) may be similar to M/S phase cells. To test this, we used RNA-seq to compare the gene expression profile of G+ and G− grown cells. This revealed 324 DEGs (Data S3). As expected, G− cells showed high expression of genes associated with gluconeogenesis and the glutamate metabolic process (e.g., fbp, pckA), rather than glycolysis. However, G− cells also exhibited changes in cell-cycle-dependent gene expression (e.g., cdc20, aurK, cdc45, top2). Most importantly, genes with peaks associated with M/S phase following cold shock release are over-represented, with a corresponding decrease in G2-associated gene expression (Figure 3B). This suggested that metabolic status affects cell cycle dynamics, with G− cells stalled in late G2 phase when M/S-associated transcription increases, thus resulting in a prestalk fate bias (Figure 3C). To confirm this, we used the single cell growth and differentiation assay to determine the lineage potential of G− grown cells at different cell cycle phases. This revealed G− grown cells with a longer than average G2 phase do exhibit a prestalk fate bias (Figure 3D), thus supporting the idea that the coupling of cell cycle phase to differentiation propensity underlies cell type proportioning in *Dictyostelium*. However, they also illustrate that cells are susceptible to extrinsic environmental perturbation of these cell cycle dynamics.

### Ras Activity Rescues the Stalk Bias Caused by G− Growth without Affecting Cell Cycle Dynamics

Developmental mechanisms should result in reproducible cell type proportioning (within normal parameter space). We, therefore, hypothesized that robustness in *Dictyostelium* development would be facilitated if extrinsic environmental fluctuations that perturb cell cycle dynamics could be buffered. Increases or decreases in buffering activity would therefore be expected to rescue or exacerbate the effects of growth in the absence of glucose, *but without altering cell cycle dynamics*. Previous studies suggested that the putative Ras-GEF *gefE* might act in this way. For example, *gefE* knockout results in a sporey bias in G+ cells, and can rescue the stalk bias caused by G− growth (Figure 4; Chattwood et al., 2013), but without affecting growth. Consequently, regulation of the levels of Ras activity provided a standout candidate for a buffering system.

The *Dictyostelium* genome encodes 11 putative Ras homologs, but only three of these (*rasC*, *rasD*, and *rasG*) are expressed at appreciable levels during multicellular developmental stages, when cell fate choice is made (Rosengarten et al., 2015). To further explore the connection between Ras signaling, cell fate choice, and buffering, we compared the effects of knocking out *gefE* with other genetic manipulations that should result in Ras pathway attenuation or hyper-activation. First, we generated *rasC*, *rasD*, and *rasG* gene knockouts in the AX3 genetic background used in this study. We found that *rasD*^−^ knockout mutant cells phenocopied the *gefE*^−^ mutant and exhibited a spore bias in chimera or forced wild-type cells to adopt stalky fates (Figure 4A). As previously reported, *rasC*^−^ mutant cells are blocked during aggregation when clonally developed (Lim et al., 2001) or when in the majority in chimera. However, this defect is non-cell-autonomous, and labeled *rasC*^−^ mutant cells efficiently enter chimeric development when mixed with a majority of wild-type cells. This revealed that *rasC*^−^ mutant cells, unlike the *rasD*^−^ mutant, do not exhibit a cell fate bias and are distributed evenly throughout chimeric slugs. Finally, we tested the effects of knocking out *rasG*. *rasG*^−^ mutant slugs are smaller than wild-type when developed clonally or in the majority. Most importantly, however, *rasG*^−^ mutant cells actually show the opposite phenotype to the *rasD* mutant, as they exhibit a stalk bias, forcing wild-type cells to adopt the prespore cell fate. This is consistent with the fact that *rasG*^−^ mutant cells express higher levels of activated RasD (Khosla et al., 2000). Alternatively, this may be because *rasG*^−^ mutant cells, like G− cells, exhibit a longer cell division time than wild-type cells (Tuxworth et al., 1997). These findings thus support the idea that Ras family members play different roles during *Dictyostelium* development and that RasD activation is dependent on GefE. Indeed, *gefE*^−^ mutant cells exhibit decreased RasD activation, whereas RasC or RasG are unaffected (Chattwood et al., 2013).

To confirm this idea, we also generated strains in which constitutively active G12T versions of RasC, RasD, or RasG were expressed under the control of a tetracycline-inducible promoter. This allowed expression to be transiently induced during growth. When labeled wild-type cells were mixed with cells transiently induced to express activated Ras, expression of constitutively active RasD^G12T^ resulted in a strong stalky fate bias (i.e., opposite to the *rasD*^−^ knockout). We also found that conditional expression of constitutively active RasG^G12T^ or RasC^G12T^ tended to push cells toward the prestalk fate. However, the effect was much weaker than that seen for RasD^G12T^. These findings support earlier studies that showed Ras proteins can exhibit a degree of functional overlap when overexpressed (Khosla et al., 2000). However, when knockout and overexpression studies are taken together, our results suggest that GefE acts through RasD to promote prestalk cell fate choice. This idea is supported by monolayer culture assays, where *rasD*^−^ mutant cells formed fewer stalk cells than wild-type, and transient activation of RasD^G12T^ increased stalk cell differentiation (Figure 4B).

Chimeric development and monolayer differentiation assays suggest that GefE specifically controls RasD to ensure correct cell fate choice. Therefore, we next tested whether these effects were due to changes in the response threshold (which is consistent with a buffering mechanism), rather than changes in cell cycle dynamics. The doubling time of wild-type cells (7.4 hr) was indistinguishable from *gefE*^−^ (7.8 hr), *rasD*^−^ (7.3 hr), or RasD^(G12T)^ (7.8 hr) cells (Figure 4C). Furthermore, we found that the lengths of M/S and G2 phases were indistinguishable in *gefE*^−^ mutant and wild-type cells (Figure 4D; Student’s t test, total: p = 0.27; M/S: p = 0.18; G2: p = 0.28), whether cells were grown in the presence or absence of glucose (Figure 4D). Together, these results suggest that nutritional history affects cell cycle phase lengths and thus the amount of time cells spend in a signal sensitive state. In contrast, the effects of altering RasD activity are due to changes in the cellular response threshold to differentiation-inducing signals. Indeed, we found that *gefE*^−^ knockout affects the responsiveness to prestalk inducing signals during all phases of the cell cycle (Figures 4E and 4F).

### Cell Cycle Dynamics and Ras-Dependent Signal Threshold Are Negatively Coupled

**Figure 5:**
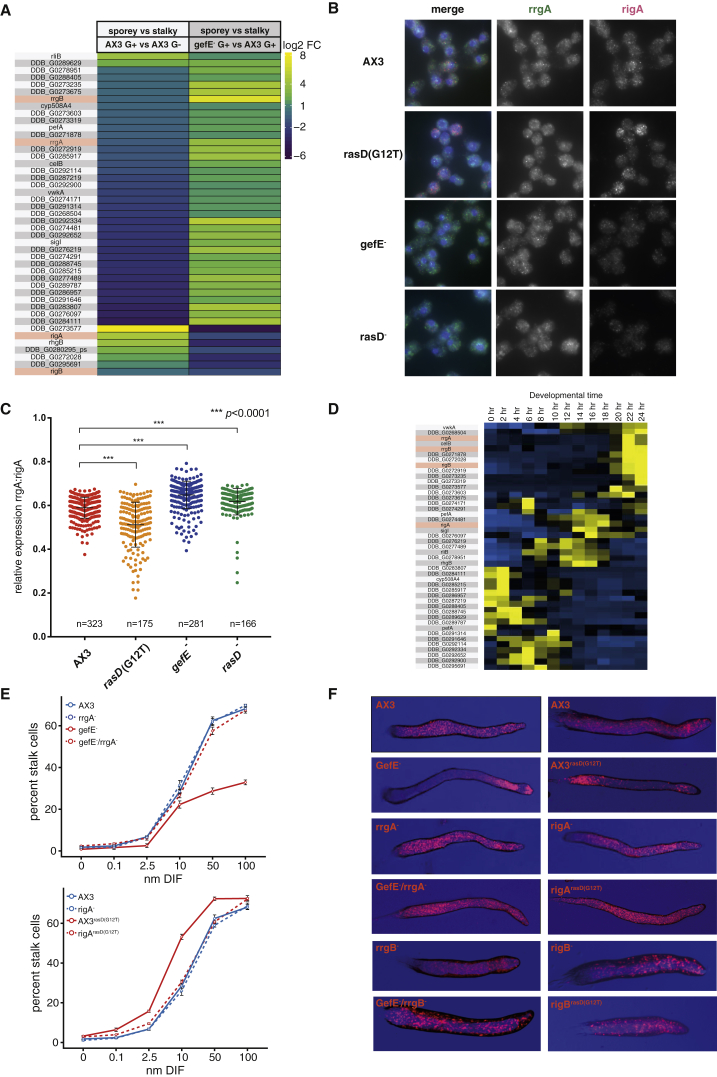
Ras Activity-Dependent Gene Expression Is Coupled to Changes in Cell Cycle Dynamics (A) RNA-seq reveals reciprocal genes expression changes in response to GefE and nutrition. RNA-seq was performed on AX3 G+, AX3 G−, and *gefE*^−^ G+ cells. Ras-dependent genes were identified by comparing gene expression of *gefE*^−^ G+ and AX3 G+ samples. A highly significant proportion of these 45 DEGs (80%) also show a >1.5 × FC in expression between AX3 G+ and AX3 G− (hypergeometric test, p = 4 × 10^−15^). Comparison of log_2_ FCs of the 36 overlapping genes shows that stalky nutritional bias results in sporey low Ras gene activity. Candidate genes are highlighted in red. (B) RNA-FISH of rrgA and rigA. rrgA and rigA are heterogeneously expressed, and expression levels depend on RasD activation. (C) Quantification of rrgA and rigA RNA-FISH. The relative expression of each gene in each cell was calculated as an index of expression rrgA/(rrgA + rigA). Values of 1 or 0 represent only rrgA or rigA expression, respectively. (D) Ras-dependent genes are maximally expressed during multicellular development. Expression profiles of RasD-dependent genes were determined from RNA-seq data at different time points during development. (E) rrgA and rigA mutant behavior in stalk cell induction monolayer assays. rrgA knockout in AX3 does not significantly affect stalk cell differentiation, whereas the *rrgA*^−^/*gefE*^−^ double mutant rescues *gefE*^−^ mutant prespore bias (see supplementary Mendeley data for p values). rigA knockout in AX3 does not significantly affect stalk cell differentiation, whereas the rigA/AX3^rasD^(G12T) strain shows significantly reduced stalk cell differentiation compared to AX3^rasD^(G12T). Plotted values are the mean of three replicates, and error bars depict the SEM. (F) Behavior of RasD-dependent gene knockouts in chimeric development. Labeled cells were developed in a 50:50 ratio with unlabeled AX3 cells. The *rrgA* and *rrgB* single mutants do not significantly affect cell fate choice. *rrgA*^−^ and *rrgB* knockouts in the *gefE*^−^ mutant rescue the tip and prespore bias seen in the *gefE*^−^ single mutant. The dictyBase: DDB_G0247655 and rigB single mutants do not significantly affect cell fate choice. The rigA/AX3^rasD^(G12T) and rigB/AX3^rasD^(G12T) strains show significantly reduced collar and back stalk cell differentiation bias compared to AX3^rasD^(G12T).

Changes in cell cycle dynamics or RasD-dependent changes in the response threshold to differentiation signals can affect cell fate choice and proportioning. Therefore, we next tested whether changes in cell cycle dynamics that increase the number of prestalk cells result in a compensatory decrease in RasD activity (i.e., favoring prespore cell differentiation) to attempt to rebalance cell fate proportioning. RNA-seq gene expression profiling was first used to identify genes associated with high RasD activity (wild-type) or low RasD activity (*gefE*^−^). This revealed 45 DEGs (Figure 5A and Data S3). We next used these genes to determine whether Ras activity was increased or decreased when cells were grown in prestalk biased G− or prespore biased G+ conditions. We found a highly significant proportion of RasD-dependent genes (36 out of 45; hypergeometric test; p = 4 × 10^−15^) were also affected by nutritional history (>1.5 × increase or decrease in expression). Most importantly, when the level of expression of these genes was compared, prestalk-biased G−-grown cells were more similar to prespore-biased low RasD *gefE*^−^ cells (Figure 5A). Therefore, these data suggest that when cells are subjected to growth in conditions that affect the cell cycle and make cells more likely to differentiate as prestalk cells, they also exhibit a gene expression profile that is associated with the prespore-biased low RasD gene state. This behavior is consistent with a cell fate buffering mechanism.

To further test this idea, we studied the behavior of representative Ras-dependent genes from the putative buffering network. rigA and rrgA were chosen because they exhibit representative (average) changes in response to *gefE* mutation and glucose (Figure 5A). Furthermore, their relatively high levels of vegetative expression in wild-type cells (Data S3) enabled us to confirm these data by different methods (see below). For example, we examined relative transcript abundance and thus *gefE*-dependent gene activity in single cells by RNA-fluorescence in situ hybridization (FISH). This revealed that the relative expression of both genes is similar in most cells (Figures 5B and 5C). However, a significant number of cells express one gene at higher levels. In wild-type, this tends to be rigA (Figures 5B and 5C). In RasD^G12T^ cells, the number of cells expressing high levels of rigA increases further, whereas in *gefE*^−^ and *rasD*^−^ mutant cells the number of cells expressing high levels of rrgA is increased (Figures 5B and 5C).

Although RNA-seq was performed on growing cells, we found that genes in the RasD-dependent gene network are maximally expressed during multicellular stages when cell fate choices are made (Figure 5D). Similarly, GO annotation reveals enrichment for plasma membrane and transmembrane proteins (p = 0.03), as well as secreted proteins (p = 0.01), which is consistent with a role in cell-cell signaling. Therefore, we next tested whether rrgA and rigA are required for normal cell fate choice. Knockout mutant lines were created in otherwise wild-type cells (i.e., “normal” RasD activity), cells in which the level of RasD activity was reduced through disruption of *gefE*^−^ or elevated through expression of constitutively activated RasD^G12T^. The behavior of these cells was tested in monolayer cell culture or in chimeric development with wild-type cells. Knockout of rrgA in a *gefE*^−^ background (which normally results in higher expression of rrgA) is sufficient to rescue the prespore cell fate bias (tip and prespore in chimeric slugs) seen in low *gefE*^−^ mutant cells (Figures 5E and 5F). Similarly, knockout of rigA in an AX3^rasD^(G12T) background (which normally results in higher expression of rigA) is sufficient to rescue the prestalk cell fate bias (collar and back in chimeric slugs) caused by expression of constitutively active RasD^G12T^ (Figures 5E and 5F). In both cases, no phenotype is seen in rrgA or rigA single mutants (Figure 5). This finding is not specific to these genes. We also generated knockouts in two other RasD-dependent genes and found their changes in expression in response to *gefE* gene disruption (*rrgB* increases, and rigB decreases) could also predict their effects on cell fate choice. Therefore, consistent with their patterns of expression, genes in this network are required for normal cell fate choice when cells are forced into low or high RasD states.

### Natural Variation in Cell Cycle Dynamics Results in Changes in Ras-Dependent Gene Expression

**Figure 6:**
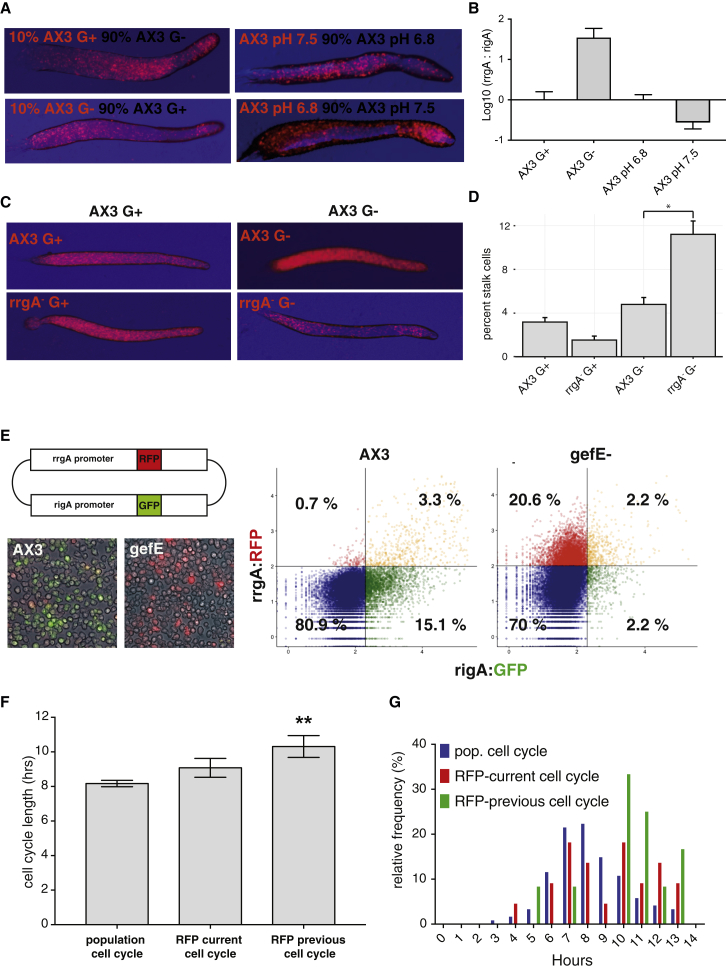
Ras Activity Buffers Extrinsic Fluctuations that Affect Cell Cycle Dynamics (A) Altering nutrition or pH during growth affects cell fate choice. RFP labeled or unlabeled cells were grown in G+ medium at pH 6.8 and 7.5 or in G− medium at pH 6.8. Chimeric development revealed cells at more alkaline pH are biased to the prespore cell fate, whereas low glucose results in a stalky bias. (B) Altering nutrition or pH during growth affects Ras network activity. Expression of rrgA and rigA was measured in G+ pH 6.8 and 7.5 and G− medium pH 6.8 by qPCR (3 replicates each). In stalky G− cells, the expression of the low Ras activity reporter rrgA increases relative to high Ras activity reporter rigA expression. This trend is reversed in sporey alkaline pH. Error bars depict SEM. (C) *rrgA*^−^ mutants are more biased to collar and back prestalk cell differentiation in chimera when grown in G−. RFP labeled AX3 and *rrgA*^−^ cells were grown in G+ or G− medium and mixed 50:50 with unlabeled AX3 G+ or AX3 G− cells. Only G− *rrgA*^−^ cells show a defect and adopt the collar and back prestalk cell fate when mixed with wild-type G− cells. (D) Increased stalk cell differentiation in *rrgA*^−^ mutant cells when grown in G−. *rrgA*^−^ mutant cells do not show a prestalk bias when grown in G+ medium, but the number of stalk cells is significantly increased compared to wild-type cells when grown in G− medium. Error bars depict SEM. (E) An rrgA and rigA dual promoter construct acts as a RasD network activity reporter. A plasmid containing the promoter of rrgA(RFP) and rigA(GFP) was transformed into wild-type and *gefE*^−^ cells. The majority of the cells (80.9% and 70%) express neither GFP nor RFP. In wild-type, the majority of fluorescent cells predominantly express only GFP (15.1%). In the *gefE*^−^ mutant, the majority of the fluorescent cells express RFP (20.6%). Only 3.3% and 2.2% express both markers, respectively. (F and G) Cells that induce rrgA expression have a longer than average cell cycle. Cell cycle length was measured in cells transformed with the rrgA promoter-RFP reporter. Cells that induced the expression of rrgA have a slightly longer cell cycle (F), while the cell cycles of the mothers of rrgA-expressing cells are significantly longer (G) (one-way ANOVA; p = 0.0019). Error bars depict SEM.

Gene expression changes in response to glucose deprivation or RasD levels suggest that RasD-dependent gene expression provides a buffering mechanism to counter the effects of extrinsic variation on the cell cycle and thus cell fate. We, therefore, tested whether these effects are specific to nutritional history or also seen in response to other environmental perturbations. For example, drugs that affect intracellular pH have previously been shown to affect cell fate choice (Gross et al., 1983, Kubohara et al., 2007). Hence, we tested whether growth at different pHs also affects cell fate choice. When compared to G+ cells grown in “normal” pH-6.8 medium, pH-7.5 cells exhibit a sporey cell fate bias in chimera. This contrasts with nutritional perturbation, where G− cells exhibit a stalky bias (Figure 6A). qPCR revealed that while stalky G− growth resulted in a sporey gene expression profile (increased rrgA versus rigA), sporey pH-7.5 growth resulted in a stalky gene expression profile (increased rigA versus rrgA) (Figure 6B).

The directionality of gene expression changes in response to pH or levels of glucose is consistent with a buffering mechanism. We therefore next tested whether these gene expression changes are required for cell type proportioning in response to changes in extrinsic conditions. Knockout of rrgA was found to have no effect when cells were grown under normal G+ conditions (Figures 6C and 6D) in chimeric development and monolayer differentiation. However, when rrgA knockout cells were grown in G− conditions (i.e., when rrgA gene expression is normally induced), clear defects were seen. In chimeric development, G− mutant cells were found to adopt the stalky collar and back fate in chimeric slugs, even when mixed with G−-grown wild-type cells (Figure 6C). Furthermore, mutant cells were hypersensitive to the effects of G− growth and showed an increased propensity to differentiate as prestalk cells in monolayer cell culture (Figure 6D).

The observed requirement for rrgA in specific growth conditions is consistent with the idea that changes in GefE/RasD activity lessen the effects of cell cycle perturbations. Therefore, we next determined whether changes in GefE/RasD network activity could also be seen under normal physiological conditions. For example, a small number of cells show a significantly longer than average cell cycle (i.e., exhibit “natural” cell cycle perturbation) even when grown in G+ conditions. These cells also have a slight tendency to become stalk cells (Figure 3C). We, therefore, tested whether they also decrease RasD network activity (e.g., increase rrgA expression) in an attempt to compensate. For this, we visualized and quantified gene network activity in living cells. A dual reporter strain was generated in which the promoters of rigA and rrgA were used to drive GFP and RFP expression, respectively. This showed similar behavior to that seen by RNA-seq and RNA FISH (Figure 6E). We next used live cell imaging to simultaneously monitor cell cycle dynamics and levels of rrgA:RFP expression in single cells. The cell cycle length of each cell that initiated rrgA:RFP reporter gene expression was measured. Because translation and folding of RFP protein are likely to introduce a delay in the system, we also measured the length of the previous cell cycle. These data were then compared to the cell cycle length of the population as a whole. Cells that activated rrgA reporter expression, and thus down-regulated Ras activity, were found to have recently exhibited a significantly longer cell cycle than the average of the general population (Figures 6F and 6G). Together, these data strongly support the idea that coupling of the signal response threshold through the Ras gene network to the cell cycle dynamics serves to buffer extrinsic fluctuations in a cell fate oscillator and maintain robust cell fate proportioning.

### A Simple Model for Cell Cycle Control of Cell Fate

**Figure 7:**
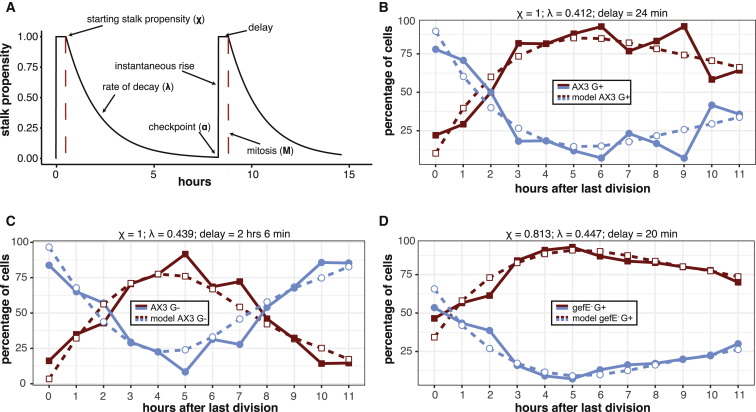
A Simple Model for Cell-Cycle-Dependent Cell Type Differentiation (A) Schematic of the model. The stalk propensity of a typical cell is illustrated through two generations under the model. Directly after mitosis (M), a cell has a starting stalk propensity (χ), which can be estimated from the experimental data. This starting propensity is then assumed to decrease with an exponential rate of decay (λ) until the cell reaches a checkpoint in the cell cycle (α). At this point, the stalk propensity increases instantaneously to its maximum value, where it is delayed by a certain time depending on extrinsic influences (e.g., nutrition). (B) Simulated data fit the experimental data from AX3 G+. A simulated cell population was generated in which all cells are asynchronously dividing (i.e., after 96 hr of growth from a single progenitor; see Figure 1G). A parameter combination of χ = 1, λ = 0.412, and delay of 24 min fit the experimental data best. (C) Increasing the delay between the checkpoint and mitosis explains the pattern seen in G− growth. A parameter combination of χ = 1, λ = 0.439, and delay of 2 hr and 6 min fit the experimental data best. (D) Decreasing the starting stalk cell propensity explains the pattern seen in *gefE*^−^ cells. A parameter combination of χ = 0.813, λ = 0.447, and delay of 20 min fit the experimental data best.

Our data suggest that a noisy cell cycle oscillator controls cell fate choice in *Dictyostelium*, while coupling of cell cycle dynamics to response thresholds confers robustness in the face of extrinsic environmental perturbation. In order to gain insights into the underlying mechanism, we used our highly quantitative data to build a model for cell cycle control of cell fate choice. The model was based on three key experimental observations. First, the probability of stalk cell differentiation is highest in cells that have just undergone mitosis. Therefore, the model assumed the existence of a cell cycle-associated factor (CCAF) that determines the stalk cell differentiation propensity (χ), with a propensity of 1, indicating that it will almost certainly adopt the stalk fate. Second, χ declines gradually following mitosis. Hence, the model assumed the breakdown of CCAF is gradual with rate λ, rather than stepped. The propensity of any cell to adopt the stalk fate was then modeled as a function of the time since mitosis (Figure 7A; STAR Methods). Finally, χ rises in cells that are at the end of G2 but is still significantly lower than cells that have just divided. Consequently, the model assumed the existence of a checkpoint at the end of G2 and that CCAF levels rise rapidly between this checkpoint (α) and mitosis. We found that the inclusion of this checkpoint α before mitosis, after which CCAF levels rise to saturation levels instantaneously and are then held constant until after mitosis, resulted in a slight rise in χ in cells with a longer than average cell cycle length (as they are more likely to be close to this checkpoint). This cell fate model could also be coupled to the previously described stochastic model that we constructed for cell cycle length variation in cell populations and thus also takes into account the observed correlation of cell cycles between sister cells. Thus, having created a population of cells using this stochastic model, we were able to assign fates to each cell based on the probability given by the propensity function in Figure 7A. Figure 7B demonstrates the excellent fit between the experimentally observed behavior of wild-type cells grown under G+ conditions and the model.

To further test the model, we addressed whether it could also predict the behavior of G−-grown cells. Experimental data suggest they exhibit a 1–2 hr block at a G2/M checkpoint, resulting in a high propensity to adopt the stalk fate. We fitted the data for G− cells to the model, which resulted in a longer delay between α and mitosis. This model reflected the observed data well, and G− cells blocked in late G2 exhibited a higher likelihood of stalk cell differentiation than G+ cells in late G2. This is because cells blocked in late G2 have a higher probability to have passed the checkpoint and reached saturating CCAF levels. The model also predicted that the rate of decay of CCAF was the same as that seen for G+ cells (Figure 7C). Finally, we also addressed whether the model could help explain why lowering Ras activity decreases stalk bias. Simply changing the amount of CCAF that accumulated at mitosis allowed the experimental data to fit the model (Figure 7D). Therefore, while this simple model may not fully capture the true behavior of CCAF, it is able to recapitulate most experimentally observed features of the cell fate system.

## Discussion

### Buffering or Mistake Correction?

Developmental systems must be robust to perturbation by extrinsic variation that affects signaling dynamics or biochemical reaction rates. In addition, intrinsic stochastic variation is an inevitable consequence of gene expression kinetics. Mistakes can be corrected through cell-cell communication if information about the relative proportions of the cell types is relayed. Our results suggest that the underlying circuits can also be buffered to ensure a robust and reproducible initial outcome. This may be because the cell cycle must exhibit stochastic variation to generate a sufficiently de-synchronized population of cells. This does, however, render the cell cycle susceptible to perturbations caused by shifts in external environmental conditions, such as nutritional availability. Here, we find that *Dictyostelium* cells have evolved an elegant solution, in which changes in the cell cycle result in compensatory changes in signal sensitivity and thus the probability of cell type differentiation (Figure S2). In this, extrinsic environmental changes that affect cell cycle dynamics trigger changes in a RasD responsive gene network. *In silico* analyses reveal this network is enriched in genes that have the potential to affect cell signaling responses, such as transmembrane and plasma membrane proteins. One of these, rigA, encodes a protein with sequence similarity to human histidine rich glycoprotein, which has been shown to modulate different signaling pathways (Jones et al., 2005). Another, rrgB, is related to components found in the yeast spindle pole body (centrosome in *Dictyostelium* and mammalian cells), which is now widely accepted to serve as a hub for the integration and coordination of signaling pathways, including cell cycle-related signaling (Arquint et al., 2014). Finally, it is interesting to note that RhgB encodes a rhesus-like glycoprotein and thus a putative ammonium transporter, given long-standing observations linking ammonium and intracellular pH in cell fate choice and responses to cAMP and DIF-1 in *Dictyostelium* (Gross, 2009).

### Linking the Cell Cycle to Cell Fate Choice

Links between cell cycle progression and cell fate decisions are well established. Recently, however, there has been renewed interest in the possibility that the *phase* of the cell cycle in which cells receive differentiation cues could affect fate choice. Much of this recent work has focused on differentiation events that occur in G1 (or indeed different phases of G1). However, a cell cycle-based mechanism has the potential to generate further cell type complexity if other phases of the cell cycle also affect fate choice. In fact, *Dictyostelium* cells have little or no G1. Instead, we find that prestalk cell differentiation is favored in S phase and early G2, and prespore cell differentiation in mid G2. It should also be noted that our studies reveal the potential for more complexity because multiple prestalk lineages are favored during S phase/early G2. It will be interesting to determine whether this is due to different windows of opportunity within this phase of the cell cycle or whether another level of regulation (perhaps stochastic) is layered on top of the cell cycle.

While our studies reveal that different phases of the cell cycle can affect lineage choice, the underlying mechanism is still poorly understood. Indeed, there are many differences between cells at different cell cycle stages, including cell size, nuclear volume, and DNA content. The activity of cell cycle regulators also rises and falls sharply as cells transition from one state to another, and these could directly affect regulators of developmental transcription. Indeed, we find that the decision of a cell to enter mitosis results in an abrupt shift in cell fate propensity. If this is due to the accumulation of a cell cycle regulatory complex, our studies will aid in its identification because they reveal the kinetics of its activity. Furthermore, due to the ease with which genes that influence developmental decisions can be isolated in *Dictyostelium* and tested for effects on the cell cycle or cell signaling, studies of this system will likely pave the way for a better understanding of this process.

## STAR★Methods

### Key Resources Table

**Table:** 

REAGENT or RESOURCE	SOURCE	IDENTIFIER
**Bacterial and Virus Strains**		

Klebsiella aerogenes	Dicty Stock Center	dictyBase: DBS0349838

**Chemicals, Peptides, and Recombinant Proteins**		

HL5 medium	Formedium	HLG0102
KK2	This study	N/A
Oxoid purified agar	ThermoFisher	LP0028B
G418	Sigma	000000004727878001
conditioned media	This study	N/A
DIF-1	ENZO life sciences	BML-GR324-0100
stalk medium	This study	N/A
EDTA	Sigma	E6758
HCR probe sets and fluorophore-labeled HCR hairpins	Molecular Technologies	N/A

**Deposited Data**		

SOLiD sequencing data AX3 G+ / G-; gefE^-^ G+/G-	This study	SRA: SAMN07834373 - SAMN07834380
Illumina HiSeq 4000 81 AX3 single cells	This study	SRA: SAMN07833758 -SAMN07833838
Datasets for Figures 1, 2, 3, 4, 5, and 6 and three additional figures	This study	https://doi.org/10.17632/rvny4rmfpp.1

**Experimental Models: Organisms/Strains**		

*Dictyostelium discoideum* AX3	Dicty Stock Center	dictyBase: DBS0235539
AX3 cells co-transformed with pspA-GFP and ecmAO-RFP	This study	N/A
AX3 cells co-transformed with *ecmB-GFP and ecmAO-RFP*	This study	N/A
AX3 cells co-transformed with *ecmB-GFP* and *pspA-RFP*	This study	N/A
AX3 cells transformed with PCNA-RFP intra-chromosomal reporter	This study	N/A
AX3 gefE^-^ cells transformed with PCNA-RFP intra-chromosomal reporter	N/A	N/A
AX3 cells transformed with dual rrgA/rigA promoter reporter construct	This study	N/A
AX3 gefE^-^ cells transformed with dual rrgA/rigA promoter reporter construct	This study	N/A
AX3 rrgA knockout	This study	N/A
AX3 rigA knockout	This study	N/A
AX3 dictyBase: DDB_G0292996 knockout	This study	N/A
AX3 dictyBase: DDB_G0281385 knockout	This study	N/A
AX3 dictyBase: DDB_G0293434 knockout	This study	N/A
AX3^rasC^(G12T)	This study	N/A
AX3^rasG^(G12T)	This study	N/A
AX3^rasD^(G12T)	This study	N/A

**Software and Algorithms**		

novoalignCS V1.04.01	NovoCraft	N/A
HTseq-count	https://htseq.readthedocs.io/	N/A
NGS QC toolkit	https://ccbr.github.io/Pipeliner/Tools/NGS_QC_Toolkit.html	N/A
bowtie2	http://bowtie-bio.sourceforge.net/bowtie2/index.shtml	N/A
R	https://www.r-project.org/	N/A
DESeq2	https://bioconductor.org/packages/release/bioc/html/DESeq2.html	N/A
M3Drop	https://bioconductor.org/packages/release/bioc/html/M3Drop.html	N/A
edgeR	https://bioconductor.org/packages/release/bioc/html/edgeR.html	N/A
ImageJ	https://fiji.sc	N/A
SAMtools	http://www.htslib.org/	N/A

### Contact for Reagent and Resource Sharing

Further information and requests for resources and reagents should be directed to and will be fulfilled by the Lead Contact, Christopher R.L. Thompson (christopher.thompson@ucl.ac.uk).

### Experimental Model and Subject Details

#### Strain Growth, Maintenance, and Development

*Dictyostelium discoideum* AX3 strains were cultured on lawns of *Klebsiella aerogenes* or in HL5 medium with (G+) or without (G−) 86 mM glucose. Cells were grown for 2–4 days for G− phenotypes. All cultures were maintained at log phase (1–4 × 10^6^ cells/ml) during this period. For development, amoebae were washed with KK2 (16.1 mM KH_2_PO_4_, 3.7 mM K_2_HPO_4_) and deposited onto KK2 plates containing 1.5% purified agar (Oxoid) at a density of 3.5 × 10^6^ amoebae/cm^2^. Plates were incubated for 14–16 hours at 22°C in a dark, moist box then removed and allowed to complete development in the light. For chimeric development, amoebae of two different genotypes, where one strain was labelled by expression of GFP or RFP, were mixed and developed together. Images were taken after approximately 16 hours to determine any sorting bias. Stalk cell differentiation in monolayers was quantified using the cAMP removal assay described in (Thompson et al., 2004a). All mutants were generated in the AX3 background used in this study by the insertion of a blasticidin resistance cassette into the coding sequence of the gene by homologous recombination. Constructs for gefE (Chattwood et al., 2013), rasC (Lim et al., 2001), rasD (Wilkins et al., 2000) and rasG (Bolourani et al., 2006) disruption have previously been described. Gene deletions were generated for rrgA (dictyBase: DDB_G0268600), rigA (dictyBase: DDB_G0274655) and rrgB (dictyBase: DDB_G0270640). For rigB (dictyBase: DDB_G0272238), the blasticidin resistance cassette was introduced at position 1,724,548 of chromosome 2. Double mutants were generated by transient expression of Cre recombinase before transformation with second knockout vector. For induction of constitutively active Ras proteins, cells were grown in the presence of doxycycline for 6 hours, before washing and plating for development.

### Method Details

#### RNA-Seq Sample Preparation and Sequencing

RNA libraries from AX3 and gefE^-^ cells grown in G+ or G- media (two replicates each) were sequenced on the SOLiDv4 platform resulting in 50bp single-end reads. For single cell sequencing, log phase cells were grown in tissue culture dishes before being harvested and resuspended at a density of 1x10^6^ cells/ml in HL5 for capture on a Fluidigm C1 flow cell using a medium size chip. This resulted in the capture of 81 individual cells, which were sequenced on an Illumina Hiseq 4000 resulting in 100bp paired-end reads (150bp insert). Raw reads from (Strasser et al., 2012) were downloaded from the Gene Expression Omnibus using accession number GSE30368 and processed in the same way as the other Illumina data.

#### Cell Type Differentiation in Low-Density Culture

AX3 cells were co-transformed with *pspA-GFP* and *ecmAO-RFP*, *ecmB-GFP* and *ecmAO-RFP, or ecmB-GFP* and *pspA-RFP* cell type specific reporters (Parkinson et al., 2009). Clonal lines were selected and maintained in 20 μg/μl G418. For quantification of cell type induction, log phase cells were seeded in 10 cm tissue dishes at a density of 1x10^4^ cells/ml in 9 ml conditioned media with or without 10nM DIF-1 (minimum of ten plates per experiment) to induce differentiation. Conditioned media was made by plating cells at a density of 1x10^6^ cells/ml in stalk medium (10mM MES, pH6.2, 1mM CaCl_2_, 2mM NaCl, 10mM KCl, 200μg/ml streptomycin sulphate) containing 5mM cAMP for 16 hours. After 16 hours, the media was collected and any cells removed by centrifugation. Freshly prepared conditioned media was prepared for each experiment performed. After 20 hours of incubation in conditioned media with or without DIF-1, cells were harvested from plates in 1ml KK2 plus 20mM EDTA. The proportion of fluorescent cells was determined by FACs (>10,000 cells per experiment). The data from all strains containing a given reporter gene were averaged, resulting in two biological replicates for each reporter construct. In addition, each induction was carried out on three separate occasions, resulting in a total of six biological replicates per cell type reporter. From this data, we were able to infer the percentage of cells that either do not differentiate or are non-transformed (100 – (pspA+ecmAO+ecmB)). For tracking of growth and differentiation, log phase cells were seeded at a density of 4x10^3^ cells/ml in 750 μl HL5 medium in a 3 cm glass bottomed imaging dish. Cells were filmed in multiple positions for 12-14 hours (i.e. until cell reached a density of approximately 1x10^4^ cells/ml) at a frame rate of one image every 5 minutes to allow cell tracking. The growth media was then removed and the cells carefully washed twice with KK2 (16.1mM KH2PO4, 3.7mM K2HPO4). 750μl of conditioned media plus 10nM DIF-1 was added to the washed cells to induce differentiation and cells were filmed for a further 16-20 hours at a frame rate of one image every 5 minutes. A final fluorescence image was captured to observe reporter gene expression. Cells were tracked by hand using the Fiji software from the beginning of the movie. The frame when a cell last divided prior to addition of conditioned media was recorded. Cells were tracked throughout the remainder of the movie and their final cell fate was recorded, as determined by reporter gene expression. The cell cycle stage at the time of induction was inferred from the amount of time between the addition of conditioned media, and the last cell division before induction. The same method was used for tracking the growth and differentiation of G- cells, but these cells were conditioned prior to the experiment by growing the cells in log phase in HL5 without glucose for 48 hours.

#### Assaying Cell Cycle Lengths

Cells were grown in G+ or G- media and imaged every 4 min for 16 hours. The time between two cell divisions was manually extracted by tracking single cells. Sister cells were tracked and their cell cycle was measured if a cell divided into exactly two cells. Mother and daughter cells were noted if the next division of the daughter cell occurred before the end of the movie.

#### Assaying Cell Cycle Phase Lengths

Cell cycle phase lengths were assessed using a PCNA-RFP intra-chromosomal reporter (Muramoto and Chubb, 2008). Cells were seeded at 1x10^4^ cells/ml in filter-sterilised HL5 with or without 75mM glucose and grown for 48 hours (growing to 3-5x10^6^ cells/ml). Cells were then diluted to 1x10^5^ cells/ml in G+ or G- HL5, added into each well of a glass-bottomed multi-chamber slide (Ibidi), and left to settle for 2 hours before imaging every 4 minutes for 16 hours using an Eclipse Ti widefield microscope (Nikon) with laser-assisted auto-focus, with illumination provided by a Precise LED light source at 25-50% maximum intensity. Images were subsequently analysed manually using NIS Elements Viewer (Nikon).

#### Assaying Ras Network Activity

To generate a dual rrgA/rigA promoter reporter construct, the promoters of each gene were amplified and cloned into the XhoI/BglII sites of either pDM323 (rigA) or pDM324 (rrgA). The pDM323- rigA:GFP construct was digested with XhoI/HindIII, and blunt end cloned into the NgoMIV site of pDM324- rrgA:RFP. Constructs were electroporated into vegetative AX3 and gefE^-^ cells, and selected with 10-20μg/ml G418. For time-lapse microscopy, the same protocol as used to follow PCNA localisation was used. For RNA-FISH, we performed single-molecule hybridization chain reaction (smHCR) using the In Situ HCR v2.0 protocol for tissue sections on slides provided by Molecular Technologies. In brief, we fixed vegetative cells in -20˚C methanol for 1 min. Fixed cells were treated with 0.5% TritonX-100/PBS for 10 min. HCR probe sets and fluorophore-labeled HCR hairpins were purchased from Molecular Technologies (moleculartechnologies.org). Samples were hybridized overnight with 2 nM of each HCR probe. HCR amplification was performed overnight with 60 nM of each HCR hairpin (conjugated to Alexa488 or Alexa594 to visualize rrgA and rigA mRNA respectively). We manually segmented the boundaries of cells and measured the mean signal intensity of Alexa488 and Alexa594 using ImageJ (NIH).

#### Mathematic Modeling

##### Stochastic Model for Cell Cycle Variation

In the model, after mitosis, the cell cycle lengths of each pair of daughters are drawn from a joint probability distribution. We denote the length of the daughters’ cell cycles τ_1_ and τ_2_ respectively. τ_1_ is drawn from a Gamma distribution with parameters a and b for the shape and rate respectively. The difference in times τ_1_ and τ_2_ is then assumed to be a mean-zero Laplacian random variable with standard deviation σ, so that

$$\tau_{1}∼Gamma\left(a,b\right),\;\left({\text{τ}}_{2}-{\text{ τ}}_{1}\right)∼Laplace\left(\sigma^{2}\right)\text{.}$$

The mean and variance of these cell cycle times can be computed to be equal to

$$E\left(\left\{{\text{τ}}_{1},{\text{τ}}_{2}\right\}\right)=\;\frac{a}{b},\;Var\left(\left\{{\text{τ}}_{1},{\text{τ}}_{2}\right\}\right)=\;\frac{2a+b^{2}\sigma^{2}}{2b^{2}}\text{.}$$

The variance σ^2^ of the sister-sister difference in cell length is set to be equal to the usual unbiased estimator of the variance found in the data. Likewise, unbiased estimators of the mean and variance of the cell cycles across the whole data set is then used in turn to ascertain the values of a and b.

##### Stochastic Model for Cell Fate

We construct a model to link the position of a cell in the cell cycle with its fate. In particular, we propose a simple function to represent the propensity that a given cell will become a stalk cell. This is a function of t, the current time, and where t_l_ and t_n_ are the times of the last and next mitosis event for the cell respectively.

$$P\left(stalk\right)=\{\begin{array}{cc} min\left\{\chi \text{ exp}\left(-\lambda \left(t-t_{l}\right)\right),1\right\} & t_{n}-t>\alpha \\ 1 & t_{n}-t\leq \alpha \end{array}\text{.}$$

The values of the parameters χ, λ and α are fitted to the observed data regarding cell fate. In particular, we aim to match the behaviour seen in each scenario presented in Figures 7B–7D. A least squares fit for χ and λ is used to match the exponential decay part of the function to the first six data points of each of these datasets. The value of α was determined by assuming all cells which have passed the checkpoint have stalk propensity equal to min(χ,1). In hours 7-11+, the proportion of cells in the last α minutes of their cell cycle was calculated, and then a value for α was determined in each case using a least squares fit to the data. The values found for each of these variables can be seen in each figure.

### Quantification and Statistical Analysis

#### RNA-Seq Re-processing

For the SOLiD data, three pre-processing steps using the SOLiD-pre-processor were included in the downstream analysis to remove poor quality sequences. Firstly, all transcripts were trimmed to 40bp from the 3’ end. Next, any sequences containing an unresolved base (-1 quality score, missing colour call) were also removed, as the sequence after this base call is likely to be wrong or ambiguous at best. In addition, sequences that contained 3 or more bases with a quality score of less than 22 were removed, as these bases are highly likely to be wrong, which would affect the reliability of the read towards the 3’ end. The resulting high quality reads were mapped against the 30.04.2012 *Dictyostelium discoideum* reference genome sequence (after masking the inverted repeat on chromosome 2) using novoalignCS V1.04.01 (parameters used: -l 25 -H 20 -r E 2 -s 5 -t 99 -n 40 -o SAM -k -c 8). Counts for each gene were then determined using HTseq-count and the option intersection-nonempty, while strand information was taken into account. For the Illumina data, paired-end reads from all samples were quality checked and filtered using the IlluQC_PRLL.pl script (v2.3) from the NGS QC toolkit and a read length cut-off of 70% together with a quality score cut-off of 20. In all samples more than 90% of the reads were retained, which were then mapped against the 30.04.2012 *Dictyostelium discoideum* reference genome sequence version (after masking the inverted repeat on chromosome 2) using bowtie2 version 2.0.0-beta5 and very-sensitive end-to-end mapping parameters requesting the best out of ten alignments. Resulting samfiles were filtered for uniquely mapped reads, sorted and converted into bamfiles using samtools (0.1.18 (r982:295)). The annotation file available at Dictybase was then used to count reads mapping to annotated genes using HTseq-count and the option intersection-nonempty, while strand information was taken into account.

#### RNA-Seq Data Analyses and Statistical Tests

Single cell data: Normalised expression profiles for each cell were correlated and cells with a low total read count (less than 10^6.2^ reads) and low average correlation to all other cells (less than 0.4) were excluded from the analysis resulting in 81 cells. Genes with low average read counts were also excluded resulting in 11,320 genes. Normalised read counts were then log transformed using DESeq2 (v. 1.16.1; and a PCA was used to identify two clusters of cells. Marker genes for each cluster (701 and 901) were identified using the R package M3Drop (v. 1.2.0) using a 1% FDR multiple testing correction and an AUC cutoff of 0.8.

AX3 vs gefE^-^ and AX3 G+ vs AX3 G-: Differentially expressed genes between AX3 samples grown in glucose rich media (G+) and glucose poor media (G-) as well as between gefE^-^ G+ and AX3 G+ samples were determined using the R package DESeq2 using an adjusted *p*-value of 0.05 and a foldchange cutoff of 1.5.

Cell cycle: Normalised read counts for the technical replicates were summed, while read counts for the biological replicates were averaged. Cell cycle profiles for each gene were determined by firstly computing the percentage of the expression for each time point and gene by dividing the normalised read counts for each time point by the sum of all normalised read counts. Then, outliers with expression of 1.5 x MAD (median absolute deviation) higher than the median were identified. Genes were clustered according to the highest outlier per gene while excluding genes with low average read counts and/or no outliers from the analysis. Over- and underrepresentation of DEGs or marker genes in specific cell cycle clusters was determined using the hypergeometric test implemented in the phyper function in R.

GO term enrichment: Enriched GO terms were identified using the statistical overrepresentation test (release 20160715) implemented on http://pantherdb.org using a *p*-value cutoff of 0.05 and the ‘GO biological process complete’ database as annotation data set.

### Data and Software Availability

Raw reads from all samples were uploaded to the NCBI short read archive (see Files S1 and S3 for accession numbers). Raw and processed data for Figures 1, 2, 3, 4, 5, and 6 were uploaded to Mendeley data (https://doi.org/10.17632/rvny4rmfpp.1).

## Supplemental Information

Document S1. Figures S1 and S2

Data S1. Read Counts and Analyses on Single Cell Data from 81 Cells, Related to Figure 1 Worksheet 1 contains the raw read counts for 13,408 genes and 96 cells obtained as described in STAR Methods. Worksheet 2 contains the same data filtered for low gene expression and low confidence cells resulting in 11,319 genes and 81 cells (see STAR Methods for details). These filtered data were then normalized and log transformed using DESeq2 (Worksheets 3 and 4; see STAR Methods for details). Worksheet 5 contains the cell identifier and the cluster affiliation (see Figure 1A). Worksheets 6 and 7 comprise the marker genes for clusters A and B and their respective AUCs and adjusted p values (computed by M3Drop, see STAR Methods), whereas Worksheets 8 and 9 contain the GO analyses for these marker genes. Worksheet 10 contains the p values for the hypergeometric tests conducted to check whether any genes with specific cell cycle profiles are over- or under-represented in the marker genes for clusters A and B (see STAR Methods).

Data S2. Read Counts and Analyses of the Cell Cycle Data from Strasser et al. (2012), Related to Figures 1 and 3 Raw reads were processed as described in STAR Methods, and read counts for all replicates separately can be found in Worksheet 1. Read counts were then normalized and counts for biological replicates averaged (Worksheet 2). Normalized read counts were converted to percentage expression per time point and clustered according to the highest outlier per gene (Worksheet 3; see STAR Methods for details). Worksheet 4 contains enriched GO terms for each time point. No GO terms were enriched in time points missing from this worksheet.

Data S3. Read Counts and Analyses for Datasets from Wild-Type and gefE− Cells Grown in G+ and G− Media, Related to Figure 5 Raw reads were processed as described in the STAR Methods, and read counts for two biological replicates per condition can be found in Worksheet 1. Normalized read counts (Worksheet 2) were then used to identify 356 and 51 differentially expressed genes between AX3 G+ and AX3 G− (Worksheet 3) and AX3 G+ and *gefE*^−^ G+ (Worksheet 4), respectively. Enriched GO terms for genes up-regulated in AX3 G+ or AX3 G− can be found in Worksheets 5 and 6. No GO term enrichment was detected in the DEGs between AX3 G+ and *gefE*^−^ G+

Document S2. Article plus Supplemental Information
