# Supplementary material for: Cell Cycle Heterogeneity Can Generate Robust Cell Type Proportioning
Source: Dev Cell. 2018 Nov 19;47(4):494–508.e4. doi: 10.1016/j.devcel.2018.09.023 (PMC6251973; doi:10.1016/j.devcel.2018.09.023)
Supplement: Document S1. Figures S1 and S2 [file mmc1.pdf]

Developmental Cell, Volume *47*

Supplemental Information

Cell Cycle Heterogeneity Can Generate Robust Cell Type Proportioning

Nicole Gruenheit, Katie Parkinson, Christopher A. Brimson, Satoshi Kuwana, Edward J. Johnson, Koki Nagayama, Jack Llewellyn, William M. Salvidge, Balint Stewart, Thomas Keller, Wouter van Zon, Simon L. Cotter, and Christopher R.L. Thompson

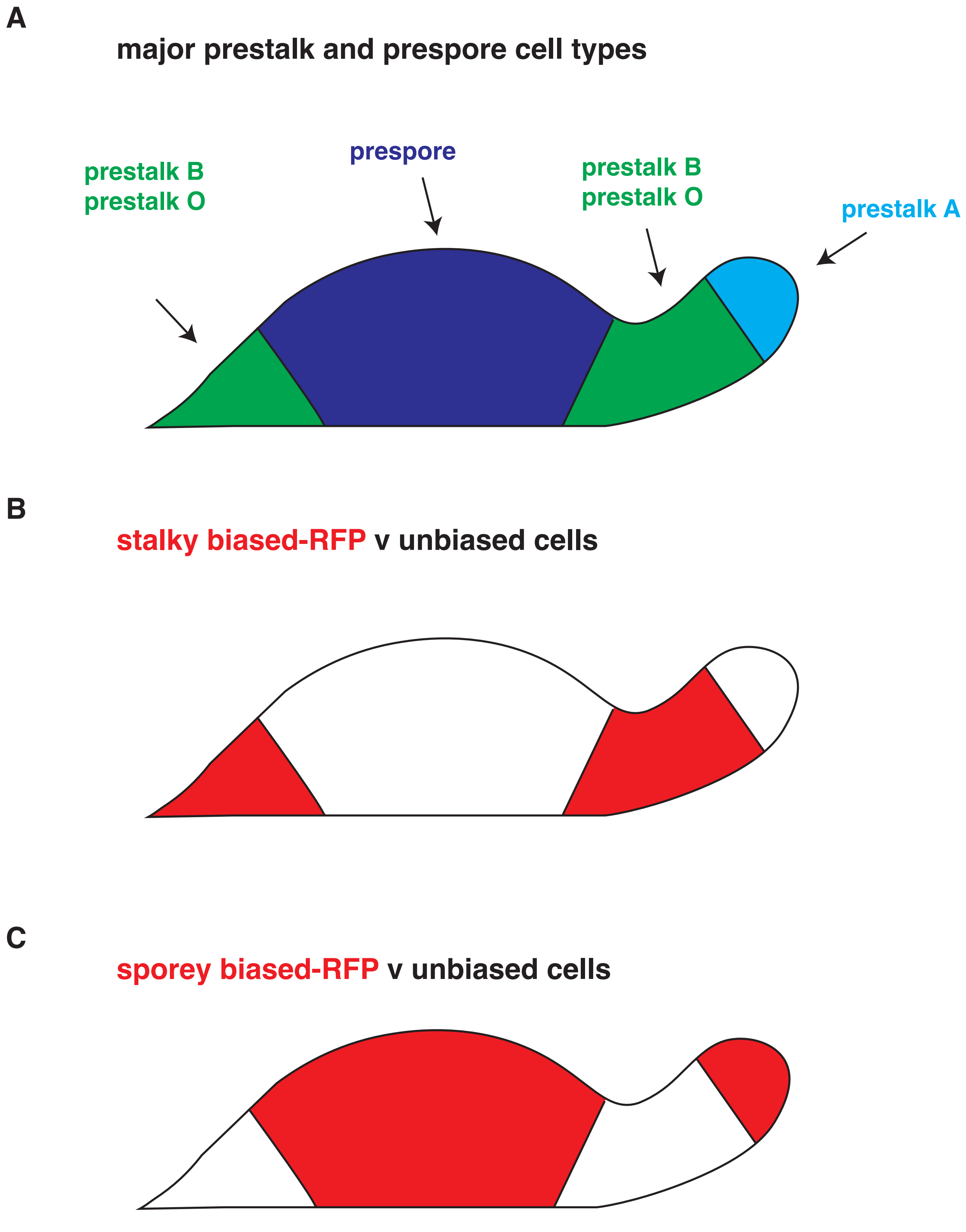

Supplementary Figure 1.
Dictyostelium cell types and behaviour of biased cells in chimera (related to introduction and all figures).

Schematic of major prestalk and prespore cell types (A). Schematic of chimeric behaviour of labelled prestalk biased cells (B) or labelled prespore biased cells (C) when mixed with unbiased cells

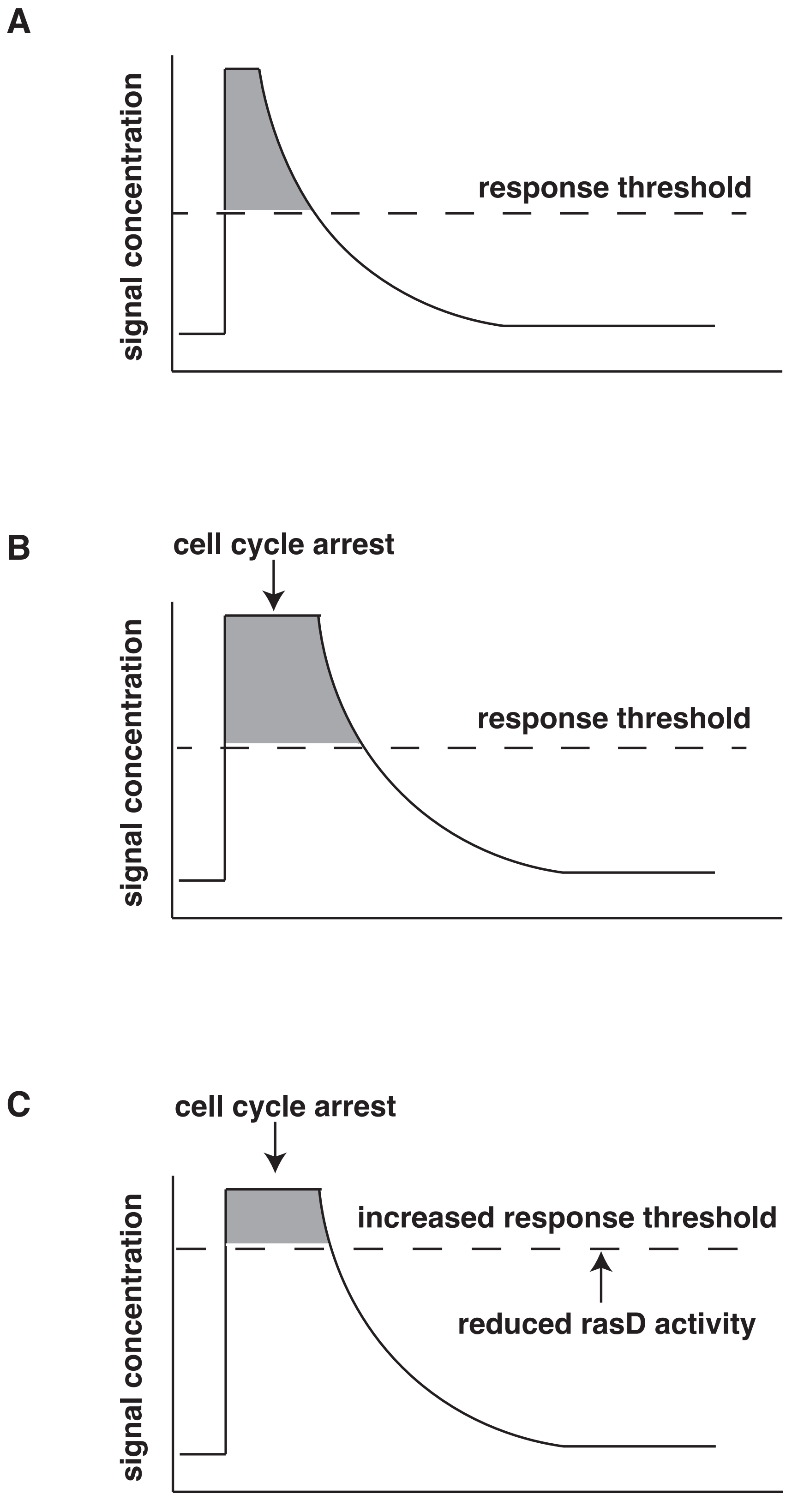

Supplementary Figure 2
Model of Ras activity buffered cell cycle dependent cell fate choice (related to figures 3-6 and discussion).

(A) Normal cells spend limited time above the threshold of responsiveness. (B) If the cell cycle is disrupted by mitotic arrest, cells would spend increased time above the threshold. (C) Cell cycle arrest is compensated by a decrease in RasD activity, which increases the response threshold.
